# A comprehensive study on anaerobic digestion of organic solid waste: A review on configurations, operating parameters, techno-economic analysis and current trends

**DOI:** 10.1016/j.biotno.2024.02.001

**Published:** 2024-02-26

**Authors:** D.Jaya Prasanna Kumar, Ranjeet Kumar Mishra, Sampath Chinnam, Prakash Binnal, Naveen Dwivedi

**Affiliations:** a Department of Chemical Engineering, Ramaiah Institute of Technology Bengaluru, Karnataka, 560054, India; b Department of Chemical Engineering, Manipal Institute of Technology, Manipal Academy of Higher Education, Manipal, Karnataka, 576104, India; c Department of Chemistry, Ramaiah Institute of Technology Bengaluru, Karnataka, 560054, India; d Department of Chemical Engineering, Siddaganga Institute of Technology, Tumkur, Karnataka, 572102, India; e Department of Biotechnology Engineering, Chandigarh University, Mohali, 140413, India

**Keywords:** Waste management, Anaerobic digestion, Co-digestion, Techno-economic study, Hydrolysis, Bioreactor

## Abstract

The excessive discharge and accumulation of solid organic waste into the environment is of severe concern across the globe. Thus, an efficient waste management system is important to mitigate health risks to humans, minimize harmful impacts on the environment, and ensure a sustainable ecosystem. The organic waste is converted into value-added products either using microorganisms or heat energy; these methods are commonly known as biochemical and thermochemical techniques. The biochemical process has the advantage of higher selectivity of the products and lower processing temperatures. The principal conversion processes of this category are fermentation and anaerobic digestion (AD). This review article focuses on AD, a potential method for treating organic waste and creating a variety of products with added value. Here we present the digestibility of various organic wastes, the role of microorganisms, the decomposition process, co-substrates, digester designs, biogas yields, by-products, environmental impacts, and overall techno-economical effectiveness of the process. Further, this review offers insights into new directions for AD for waste treatment and future research without compromising the overall feasibility and environmental sustainability.

## Highlights

• A variety of organic waste can be treated biologically by anaerobic digestion.
• Biogas can be produced at a minimal cost by co-digesting several substrates.
• Effect of process parameters on CH_4_ and H_2_ yield discussed.
• Challenges, limitations and future scope of this technology was addressed.
• Techno-economic analysis of AD is reviewed and discussed in details.

## List of abbreviations

Short Name: Full name
AD: Anaerobic digestion
TEA: Techno-economic analysis
GHGs: Greenhouse gas
OFSW: Organic fraction of solid waste
OLR: Oxygen loading rate
HRT: Hydraulic retention time
TM/HMs: Toxic metals/heavy metals
OSW: Organic solid waste
LFA: Long fatty acids
ACD: Anaerobic co-digestion
C/N: Carbon to nitrogen ratio
ABR: Anaerobic bioreactors
TAN: Total ammonium nitrogen
MC: Moisture content
BOD: Biochemical oxygen demand
COD: Chemical oxygen demand
SPAC: Spiral automatic circulation
UAFB: Up-flow anaerobic fixed bed
TVFA: Total volatile fatty acids
VS: Volatile solids
SRT: Solid retention time
SAnMBR: Submerged Anaerobic Membrane Bioreactor
VFA: Volatile fatty acids
FA: Free Ammonia
TSR: Two-stage reactors
OFMSW: Organic fraction of municipal solid waste
VOCs: Volatile organic compounds
FWL: Food waste leachate

## 1 Introduction

**Fig. 1:**
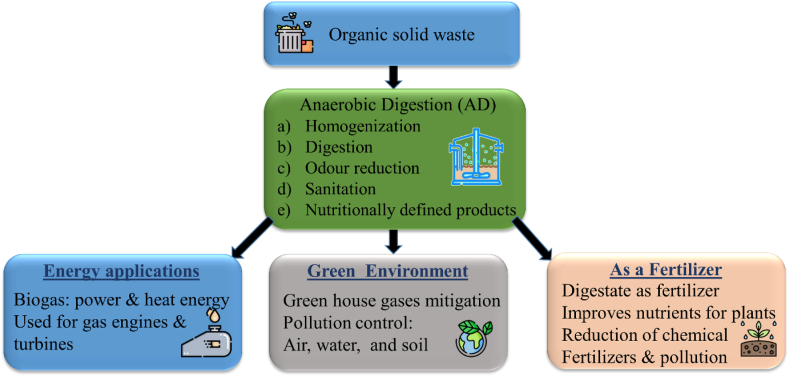
Schematic depicting the applications of AD contributing to energy, environment, and as a fertilizer.

The valuable resource contained within the solid waste (organic component) can now be transformed into valuable products through microbial processes.^1^ Anaerobic digestion (AD) is a promising technique for processing organic waste, compared to other methods, including thermal, biological, and chemical approaches.^1^ AD comprises of series of four biochemical reactions, i.e., hydrolysis, acidogenesis, acetogenesis, and methanogenesis.^1^ AD of organic waste from landfills releases CH_4_ and CO_2_ thereby causing environmental pollution.^2^ However, under controlled conditions, such as temperature, pH, nutrient composition, and retention time the AD ensures optimal microbial activity and overall process performance thereby producing value-added products like biofuel and many commercial chemicals in the absence of oxygen.^2^ The CH_4_ and H_2_, produced through this process, are considered as cleaner potential fuels than fossil fuels.^3^ Moreover, this approach does not rely on fossil fuels for energy consumption, providing an added advantage.^4^ AD is a promising technique to mitigate environmental pollution which also produces biogas and organic fertilizer a favourable material for farming. However, this method has some drawbacks such as longer retention times and the production of ammonia harms the microbial activity.^5^ Recent developments in bioreactor design have improved the use of AD for the treatment of solid organic waste and the production of many value-added products.^6^ Several innovative bioreactor designs have been developed, allowing AD to occur faster than conventional methods. In addition to AD, gasification, fermentation, pyrolysis, and liquefaction are the commonly used technologies to address environmental issues related to waste and waste management and also to promote the production of sustainable energy.^2^^,^^7^ AD has been recognized as a sustainable approach to generating renewable energy from various organic materials^8^ and recognized as a more reliable process than composting, gasification, pyrolysis, incineration, and torrefaction owed to its techno-economic feasibility.^9^ Furthermore, AD has a lower impact on air quality when compared to combustion-based methods, and it helps reduce carbon emissions by generating energy that can replace fossil fuels.^10^ The implementation of AD technologies for reducing greenhouse gas (GHGs) emissions could play a significant role in achieving the objectives of the 2016 Paris agreement, which aims at reducing the global average temperature well below 2 °C. Biogas and solid sludge produced by microorganisms upon digesting organic waste are the most viable alternatives to fossil fuels and bio-fertilizers.^11^ The AD process can utilize any organic waste, although pH and temperature can impact gas production. Fig. 1 depicts the potential of the AD pathway to produce energy from the organic fraction of solid waste (OFSW).^12^ These biogas plants play a vital role in minimizing environmental impacts by harnessing the waste to produce methane gas, such as global warming effects.^13^ Additionally, the AD process offers a practical and reliable solution to waste management, energy recovery, and nutrient recycling. For instance, biogas is produced from organic waste, and the byproduct digestate can be used as organic-fertilizer in lieu of chemical fertilizer.^14^ These organic fertilizers are known to improve soil structure, retain nutrients, permits carbon fixation, improves yield, and enhanced ability of crop to absorb water.^15^ The success of the AD relies on readily available degradable waste, access to best technology to maximize the biogas yield and other value-added products, and lower production cost.^14^

Temperature, OLR, HRT, substrate type and concentration, moisture level, ammonia, nitrogen, pressure, and pH are the common variables that influence the performance of the AD process.^16^ This article provides a general overview of the potential of AD to turn wastes into valuable products. However, it is essential to note that specific waste components, such as toxic metals/heavy metals (TM/HMs) and recalcitrant organic compounds, do not undergo degradation and may accumulate in the residue. It is important to consider this factor and must be addressed while implementing AD and it is beyond the scope of this review article.

This article presents a comprehensive review of the potential of using organic solid waste (OSW) as a sustainable source for bioenergy production, focusing on circular bio-economy. Here, we elaborate on various feeds derived from bio-waste that can be efficiently used for biofuel generation. One of the critical contributions of this review is the overview of bioenergy production strategies that have not been comprehensively (excluding a few that are majorly focused on kitchen and food waste) examined in the previous research. OSW bioenergy production has significant potential as a sustainable and circular solution to waste management and energy creation.^17^ Additionally, this article emphasizes the impact of various factors such as types of feed, the decomposition steps involved in organic solid waste (OSW) degradation, and the variability of biogas yield and techno-economic analysis (TEA). While many factors have been extensively studied, the pressure, mixing, nitrogen uptake, and NH_3_ loading have not received as much attention. This review article delineates valuable insights into OSW as a potential and sustainable bioenergy source for sustainable bioenergy production (biogas or fertilizers etc.), highlighting the significance of circular bio-economy principles in attaining sustainable development.

## 2 Variety of feedstock for bioenergy generation

**Fig. 2:**
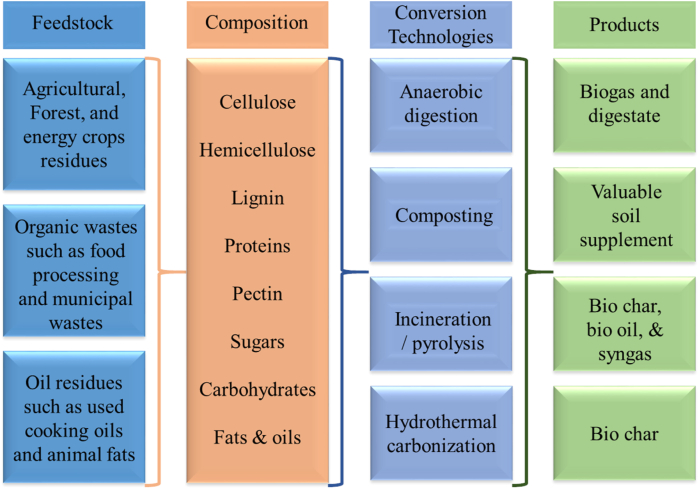
Schematic of feedstock, composition, conversion technologies, and products from the organic solid waste.

**Fig. 3:**
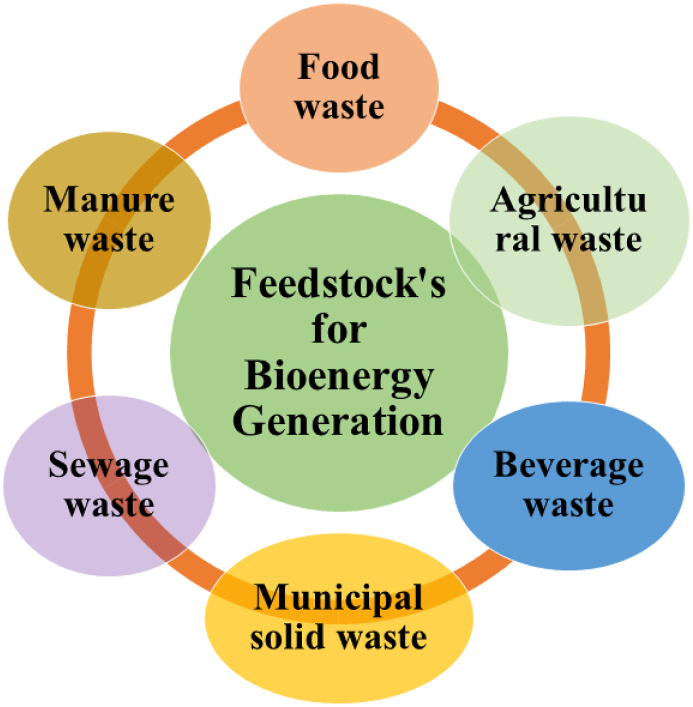
Types of waste used for bioenergy production (redrawn by Kumar et al. (2023).^21^

Food and agricultural waste contribute significantly to OSW. Improper discharge of these solid waste has adverse effects on the environment. The decomposition of organic matter in landfills also creates H_2_S and organic mercaptans, which can give rise to odour-related issues.^18^ Moreover, releasing volatile compounds into the atmosphere from landfills can cause air pollution. Surface water can percolate into the earth and pollute groundwater quality when different industrial solid waste is dumped in landfills. The quality of the air, water, and soil can all be negatively impacted by dumping solid waste in landfills. In addition, the leachate produced by the breakdown of municipal solid waste comprises toxic heavy metals and organic pollutants.^19^ It is important to understand that dumping and burning of OSW do not ensure recovery of essential elements for potential applications and the microbial degradation of the OSW can release greenhouse gases (GHGs).^20^^,^^21^ Similar studies have shown landfills in the United States rank as the third most significant source of methane production.^22^ Table 1 summarizes the primary harmful impacts of bio-waste on the environment. Composting is another extensively researched approach that can produce bio-fertilizers for farmers.^23^ To minimize the harmful effects of OSW on the environment, it is essential to promote appropriate waste management techniques. Conventionally these wastes are treated using matured technologies such as incineration, composting, landfills, and livestock feed.^24^ Table 2 presents various sources of bio-waste for the generation of sustainable bioenergy. Fig. 2 illustrates the feedstock, composition, conversion technologies, and ultimate products. Fig. 3 illustrates the assorted feeds employed for a variety of applications.

**Table 1:** The adverse impact of bio-waste on sustainability and the environment.

Source of bio-wastes	Impact on environment and sustainability	References
Food waste	Many dangerous greenhouse gases are released during the breakdown of food waste.	^25^
Crop residue	Wastes from the agricultural and industrial sectors are nutrient-rich and act as breeding habitats for microorganisms when discharged into the environment.	^26^
Fish waste/Aquaculture	Aquaculture nutrients and fish waste breakdown cause eutrophication in bodies of water.	^27^
Brewery	Brewery waste has a genotoxic impact, damaging terrestrial and aquatic creatures' DNA.	^28^
Poultry waste	Source of heavy metals, medications, bacteria, viruses, and parasites.	^29^
Municipal solid waste	emissions of hazardous gases and greenhouse gases.	^30^
Sewage	Sewage with high heavy metal content bio-accumulates in the food chain.	^31^
Domestic waste waster	Water bodies become eutrophicated due to home wastewater's high phosphorus content.	^32^
Manure and Faecal waste	Animal faeces and dung have been found to include bioactive substances, antibiotics, and heavy metals. These substances may enter the food chain and result in bio-magnification.	^33^
Sugar Industry/distillery	Large amounts of organic matter, nutrients, and metals in the wastewater generated by the sugar and ethanol industries hurt aquatic and terrestrial ecosystems.	^34^
Livestock	Farms with cattle discharge their effluents containing nitrogen and phosphorous can cause water pollution.	^35^
Paper and Pulp Industry	After washing, bleaching, and pulping, numerous dangerous chemicals are discharged as primary and secondary effluents, which causes air pollution.	^36^
Agricultural industry	Through agricultural waste, chemicals, including arsenic, benzene, mercury, and trichloroethylene, enter the atmosphere and cause respiratory and brain illnesses.	^37^

**Table 2:** Types of bio-waste, their source, and specific examples.

Types of waste	Waste production source	Examples
Municipal wastes (organic only)	Post-processing	Clothes, biodegradable residue, sewage sludge, etc.
Forestry wastes	Residue or fresh	Wood chips, bark, sawdust, wood waste from mills, and other materials.
Agriculture wastes	Harvesting and post-processing residue	Stem, straw, husk, cobs, bagasse, roots, leaves, etc.
Pulp and paper wastes	Raw post-processing residue	Lime slacker grits, boiler and furnace ash, lime mud, green liquor, wood processing residue, etc.
Food or kitchen wastes	Post-processing residue	Pulp, skin, peels, and seeds
Animal wastes	Post residue	Manure, animal residue, fish waste, dead bodies waste.

## 3 Anaerobic digestion (AD)

**Fig. 4:**
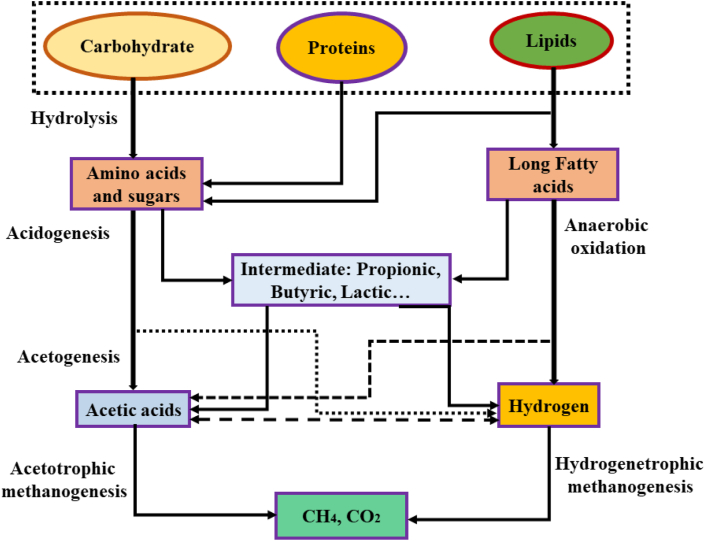
Decomposition of organic waste into final products.

The advent of inventive designs for pilot and commercial plants to treat OSW by AD gained worldwide attention in late 1990.^16^ This process utilizes diverse microbial populations to biologically convert almost any organic waste into biogas and energy-rich organic species without oxygen.^16^ The anaerobic decomposition process consists of a series of biochemical reactions such as hydrolysis, acidogenesis, acetogenesis, and methanogenesis. Compared to aerobic processes, AD is a more feasible technology for organic waste treatment and simultaneous renewable energy production, as it requires less energy and produces less sludge.^38^ Additionally, this approach helps to reduce pollution by minimizing the release of CO_2_ into the atmosphere while utilizing methane releases as potential fuel.^38^ However, AD also has some drawbacks, such as long retention times, low processing capacity, and lower removal rates of organic compounds.^38^ Fig. 4 illustrates the breakdown of waste into desirable end products; carbohydrates, proteins, and lipids are converted into smaller amino acids, sugars, and long fatty acids (LFA) via hydrolysis. Amino acids and sugars are converted into acetic acid, while fatty acids are transformed into hydrogen via acidogenesis, acetogenesis, and Anaerobic oxidation. Finally, through acetotrophic methanogenesis and hydrogenetrophic methanogenesis reactions, these compounds are ultimately transformed into CH_4_ and CO_2_.

### 3.1 Hydrolysis

In the hydrolysis stage of AD, microorganisms such as *Bacteroides, Clostridia, Bifidobacteria*, and *sometimes Streptococci and Enterobacteriaceae* break down complex bio-polymeric compounds like lipids, carbohydrates, and proteins into water-soluble compounds.^39^ This process can be slow when solid materials are used as a substrate, which can limit the overall digestion rate. Equation (1) shows that cellulose (C_6_H_10_O_5_) can be hydrolyzed into glucose (C_6_H_12_O_6_) and hydrogen (H_2_) with the assistance of either homogeneous or heterogeneous acids, resulting in the production of fermentable monosaccharide (C_6_H_12_O_6_). In the next phase, fermentative microorganisms utilize the resulting products (C_6_H_12_O_6_ and H_2_) to produce higher-chain organic compounds like volatile fatty acids.^40^

(1) $${\left(C_{6}H_{10}O_{5}\right)}_{n}+nH_{2}O\overset{\;}{\to }n\left(C_{6}H_{12}O_{6}\right)$$

### 3.2 Acidogenesis

The 2nd phase of AD called the fermentation stage, is characterized by the activation of acidogenic bacteria such as *Streptococcus, Escherichia*, *Staphylococcus, Pseudomonas, Bacillus, Sarcina, Desulfovibrio, Lactobacillus*, etc..^41^ These bacteria play a crucial role in breaking down amino acids, lipids, and glucose into volatile fatty acids, organic acids, CO_2_, and H_2_ gas, as shown in Equations (2), (3), (4), (5), (6), (7).^40^ Among the various organic acids produced during this stage, acetic acid is of the utmost significance as it acts as a substrate for methanogenic microorganisms.^42^ It is important to note that the production of volatile fatty acids is favoured when the pH is above 5, whereas ethanol production (CH_3_CH_2_OH) is favoured at a pH less than 5. The reactions halt when the pH drops below 4.^43^

(2) $$C_{6}H_{12}O_{6}\overset{\;}{\to }2CH_{3}CH_{2}OH+2CO_{2}$$

(3) $$C_{6}H_{12}O_{6}+2H_{2}\overset{\;}{\to }2CH_{3}CH_{2}COOH+2H_{2}O$$

(4) $$C_{6}H_{12}O_{6}\;\to \;3CH_{3}COOH$$

(5) $$C_{3}H_{7}O_{2}N+2H_{2}O\;\to \;C_{2}H_{4}O_{2}+NH_{3\;}+CO_{2}+2H_{2\;}+ATP$$

(6) $$C_{4}H_{9}O_{3}+NH_{3}+CO_{2}+H_{2}+ATP$$

(7) $$4CH_{3}COCOO^{-}+4H_{2}O\;\to \;5CH_{3}COO^{-}+2HCO_{3}^{-}+3H^{+}$$

### 3.3 Acetogenesis

Reactions are reversible at this stage and result in the relief of hydrogen gas. Volatile fatty acids such as acetic acid, propionic acid, and butyric acid are converted into carbon dioxide gas, hydrogen, and acetate, as depicted in Equation (8) -(9). A few examples of active bacteria in this stage are *Clostridium, Syntrophomonas wolfeii*, and *Syntrophomonas wolinii*.^41^ In the presence of water molecules, the volatile fatty acids undergo conversion to form other desirable compounds. Here water molecules serve as electron sources formed from the previous stages of AD. Equation (9) is critical because it converts the ethanol into acetate and hydrogen, which are used in the subsequent stages.^44^ The efficiency of biogas production largely depends on this stage. Acetate, which accounts for approximately 25% of the products formed undergoes a reduction of 70% of CH_4_ and 1% of H_2_.

(8) $$C_{6}H_{12}O_{6}+2H_{2}O\;↔\;2CH_{3}COOH+2CO_{2}+4H_{2}$$

(9) $$CH_{3}CH_{2}OH+H_{2}O\overset{\;}{\to }CH_{3}COO^{-}+2H_{2}+H^{+}$$

### 3.4 Methanogenesis

Methanogens are bacteria that convert acetic acid into CH_4_ and CO_2_ during the last methanogenesis stage. These organisms are highly sensitive to oxygen. The reaction between CO_2_ and hydrogen, the decarboxylation of ethanol, also produces CH_4_. This stage utilizes two types of bacteria: *acetophilic methanogenic*, which include *Methanosarcina and Methanosaeta* as the active species, and hydrogenophilic methanogenic, which is dominated by *Methanospirilum, Methanobacterium formicicum, Methanoplanus, and Methanobrevibacterium*.^41^ The former bacteria convert acetate into methane via decarboxylation, while the latter produces methane by reacting with CO_2_ and H_2_.^40^ The end product of AD is biogas, a mixture of CH_4_ and CO_2_.

(10) $$CH_{3}COOH\;\to \;CH_{4}+CO_{2}$$

(11) $$CO_{2}+4H_{2}\;\to \;CH_{4}+2H_{2}O$$

(12) $$2CH_{3}CH_{2}OH+CO_{2\;}\;\to \;CH_{4}+2CH_{3}COOH$$

## 4 Anaerobic co-digestion (ACD)

Co-digestion (co-fermentation) is a technique used for treating waste that combines innumerable waste to enhance the AD of solid organic waste.^45^ This method has several advantages, including the dilution of toxic substances, an increase in the biodegradable organic matter load, an enhancement in nutrient balance, a synergistic effect of micro-organisms, and a higher biogas yield. In addition, co-digestion provides excess nutrients that can accelerate biodegradation through bio-stimulation, increase the digestion rate, and enhance stabilization.^45^ Also, co-digestion ensures consistently stable anaerobic reactor performance, improved quality of the digestate, and increased biogas yield.^46^ Co-digestion reduces nitrogen content, improving the C/N ratio in the bioreactor feed and leading to enhanced stability. The solid waste with low nitrogen and lipid content when co-digested exploits the complementary characteristics of wastes, lowers the formation of volatile carbon compounds and NH_3_ concentrations, in turn favours the enhanced production of biogas.^47^ Researchers have demonstrated that various mixtures of agricultural, food, dairy, municipal, and industrial wastes can be efficiently co-digested (Table 3). As an illustration, studies comparing the co-digestion of municipal solid waste and industrial sludge at a 1:2 ratio to municipal solid waste alone have shown enhanced methane gas output. The maximum amount of CH_4_ was produced in a two-phase AD system when solid waste and wastewater from olive mills were co-digested.^48^ The digestate from the co-digestion process is a valuable material that can be effectively used as a soil fertilizer after minor treatment. The temperature, co-substrate concentration, inoculum ratio, and C/N ratio are the most critical factors that affect the overall efficacy of the AD process.^49^

**Table 3:** Co-digestion of various solid organic waste and their outputs.

Co-Digestion mixture	Methane yield (mL CH_4_/g VS)	C/N ratio	Temperature (^o^C)	Hydraulic retention time (days)	Digester Type/Volume (mL)	Reference
FW and waste-activated sludge	407	–	35–55	302	CSTR/3000	^50^
FW, cattle manure, and corn straw	500	13–43	35–39	25	Semi-continuous/300	^51^
FW, newsprint paper, and branches	129.70–534.40	–	37	30	Batch system/400	^52^
SS with crude or pre-treated glycerol	45	6.42	30	15–30	Three stages/100	^53^
FW and brown water	728	–	37	15–20	Two-stage digester/10,000–35,000	^54^
FW and SS	305.44	14.5	35	–	Membrane bioreactor/15000	^55^
Municipal solid waste and SS	571–675	10.60–31.40	37	45	Glass bottles/100	^56^
SS and glycerol	37–483	–	36–38	40	Continuous Operation/160	^57^
Excess Sludge with chicken manure	82.40–123.10		37–55	40	Batch system made of glass	^58^
Dark fermentation of SS and agricultural residue	52.05	14.24	35	20	Batch systems/120	^59^
Palm oil mill effluent with decanter cake	515	10.70–15.80	36–38	35	Graduated cylinder/250	^60^
Cheese whey and septage	342.22	34	35	35	Glass flask, 100	^61^
Taihu blue algae with swine manure	212.70	5.8–11.40	37	22	400	^62^
Fresh vinegar residue and Pig manure	233.80	14.5–24.40	33–37	20	Semi-continuous stirred tank reactor, 70,000	^52^
Leather waste with raw and wheat straw	43.15	10.88–68.87	35	274	Bench scale bioreactor in a cylindrical vessel, 300	^52^
Mixed microalgae and FW	640	15.43	35	40	120	^63^
Decanter cake and empty fruit bunch	257	9.70–49.50	35	35	Batch reactor, 250	^64^
Press mud and bagasse from sugar mill	450	13	35–37	35	Glass flask, 1000	^65^
Wastewater grown algae-bacteria polyculture biomass and cellulose	323–380	5.67	34.5–35.50	60	Serum Bottles/150	^66^
Rice wastewater with cow dung slurry	292	23.70	35–39	4	Two-stage/250, 500	^65^
Sargassum-pig manure	441.47	16.8	36–38	100	Serum Bottles/305	^67^
	High C/N ratio					
Corn Stover: Swine manure	281	25	36–38	40	8000	^68^
Wheat straw with cattle manure	254.60	16.60–88.10	36–38	35	1000	^69^
Banana stem and swine manure	357.90	–	36–38	40	Solid state/1000	^70^
NaOH-treated biphasic olives with FW	503.60	–	37	30	Stirred tank reactor, 6000	^71^
Rice straw and pig manure	235.80	35–38	37–55	190	Two reactors, 1000-8000	^72^
Mango and microalgal residue biomass	204.40	–	37	30	Glass bottle, 250	^73^

FW = Food waste, SS = Sewage sludge.

## 5 Anaerobic bioreactors

Anaerobic bioreactors (ABR) have shown potential for quickly digesting OSW to reduce environmental burden than conventional sanitary landfills.^74^ A bioreactor's design strongly influences a digester's performance.^42^ An efficient design must be able to continuously process a high organic load rate, achieve a short hydraulic holding time, and maximize methane production. Many ABRs used include batch reactors, single-stage continuously operated systems, and two-stage or multi-stage operated systems. Batch reactors are simple and inexpensive, enabling quick digestion and easy assessment of digestion rates. However, they have drawbacks such as unstable gas yield and quality, loss of biogas during emptying, and limitations with bioreactor heights.^75^ Single-stage continuously operated systems treat all biochemical reactions in a single reactor, while in two-stage or multi-stage continuously operated systems, a series of reactions such as hydrolysis, acidogenesis, acetogenesis, and methanogenesis occurs in various units.^76^ The two-stage ABR is considered beneficial for treating organic wastes with enhanced efficiency in biogas production and degradation of organic waste, as this method has the provision for producing the requisite bacteria and its growth in each phase. Acidogenic bacteria break down complex organic materials into volatile fatty acids and alcohols, which are then transformed into CH_4_ and CO_2_ by methanogens or archaea.^77^ The two-stage system offers the advantage of optimizing each stage independently, leading to increased stability and efficiency of the bioreactor. The acidification phase can be regulated by optimizing the hydraulic retention time, inhibiting overloading and the accumulation of toxic materials, which can negatively impact biogas production. Moreover, optimizing the biomass feed ratios and process conditions at each stage ensures the maximum amount of biogas is produced while minimizing any potential negative environmental effects. This system also enhances process stability by regulating the acidification phase through hydraulic retention time optimization, thereby preventing overloading and the accumulation of harmful substances. In summary, optimizing each stage independently in the two-stage system can increase the process stability of biogas production and minimize any adverse environmental impacts.^77^ Various bioreactors can be utilized for waste treatment. Additionally, several types of methanizers can be used to treat different types of waste.^78^ Bioreactors can be classified into wet or dry solid waste digesters based on their total solids content. Waste whose total solids (TS) content is less than 16% is considered wet, while if TS content is between 22 and 40% is considered dry.^79^ There is an intermediate category between these two, known as semi-dry. However, by Karagiannidis and Perkoulidis (2009), dry systems account for 30–40% dry matter, while wet systems account for 10–25% dry matter.^80^ It is important to note that dry organic waste (agricultural residues) has high carbon and low nitrogen content, and addition of nitrogen supplement is essential to maintain optimum carbon-to-nitrogen (C/N) ratio for improved digestor efficiency. Pre-soaking of the dry waste is another vital factor to be considered to enhance the microbial activity. The semi-dry organic waste (agricultural residue) also demands nitrogen supplement for optimum C/N ratio. The wet waste (food and agricultural residue) usually contains desired amount of C/N ratio; however, a careful monitoring of this ratio is necessary for enhanced digestor efficiency.

## 6 Biogas yield from AD of OSW

The yield of biogas from OSW typically involves the participation of several anaerobic bacteria.^80^ Table 3 shows that AD of the OSW can yield substantial biogas. Typically, biogas consists of 48–65% CH_4_, 36–41% CO_2_, up to 17% N_2_ less than 1% O_2_, 32–169 ppm H_2_S, and other trace gases.^81^ In contrast to fossil fuels, biogas has a relatively lower impact on acid rain, the greenhouse effect, and ozone depletion.^82^ AD has the potential to contribute significantly to meeting the energy needs of future generations, primarily due to its low environmental impact. Further, various factors affect biogas yield, including substrate composition and type, microbial composition, temperature, moisture, and bioreactor design. A study on the biodegradation of MSW witnessed the beginning of the methanogenic phase succeeded on the 63rd day, and the highest CH_4_ yield was observed at 70% moisture level. However, fruit and vegetable waste showed a decline in biogas production due to rapid acidification, leading to decreased pH within the bioreactor.^83^ The production of higher levels of volatile fatty acids during anaerobic digestion from waste can decrease the activity of methanogenic bacteria. However, adding co-substrates like slaughterhouse wastewater and activated sludge to fruit and vegetable waste enhances biogas yield by 52%.^84^ Behera et al. (2010) carried out investigations using simulated landfill bioreactors (lysimeters) to produce methane from food waste leachate (FWL) with a variety of inoculum-substrate ratios (ISRs). The lysimeter with an ISR of 1:1 had the highest methane yield. They suggested that liquid organic waste can be treated in bioreactors equipped with effective gas recovery and leachate collection systems..^85^ Lee et al. (2009) proposed that AD of FWL in bioreactor landfills or anaerobic digesters with proper alkalinity and salinity control could be an effective and sustainable way to convert biomass into biogas while achieving high biodegradability potential.^86^ AD relies on diverse microorganisms to break down complex macromolecules into simpler compounds.^42^ To optimize the process, it is crucial to carefully consider the waste-to-inoculum ratio and choose a suitable source of inoculum.^87^ While sludge is used as a starter culture, natural or synthetically mixed strains of microorganisms can also be employed, along with cell aggregates such as flocs, biofilms, granules, and mats.^88^ Various microbial populations have been identified to play a crucial role in AD. Ike et al. (2010) reported that microorganisms such as *actinomyces, Thermomonospora, Ralstonia, and Shewanella* break down food waste into VFA, while *Methanosarcina* and *Methanobrevibacter or Methanobacterium* are primarily a reliable source for methane production.^89^ The hydrogenotrophic species proficiently utilize H_2_ and CO_2_ other microorganisms produce to break down organic matter to generate methane. A study by Trzcinski et al. (2010) used denaturing gradient gel electrophoresis and DNA sequencing to gain valuable insights into the active microbial species involved in methane synthesis during AD ^90^ *Methanobrevibacter* spp.*, M. formicicum*, and *Methanosarcina* spp. Were identified as the primary hydrogenotrophic species responsible for methane production.^90^ Organic acids such as acetic acid (>5000 mg L^−1^) and butyric acid (>3000 mg L^−1^) can inhibit microbial activity, leading to reduced methane and energy-rich compound production during AD ^90^ Therefore, it is crucial to monitor the concentrations of organic acids in the bioreactor to ensure optimal microbial growth and energy production. Some of the methods in reducing organic acid concentrations include adjusting substrate composition, improving reactor mixing, and employing pre-treatment steps to minimize the formation inhibitory compounds.

## 7 Factors affecting AD

**Fig. 5:**
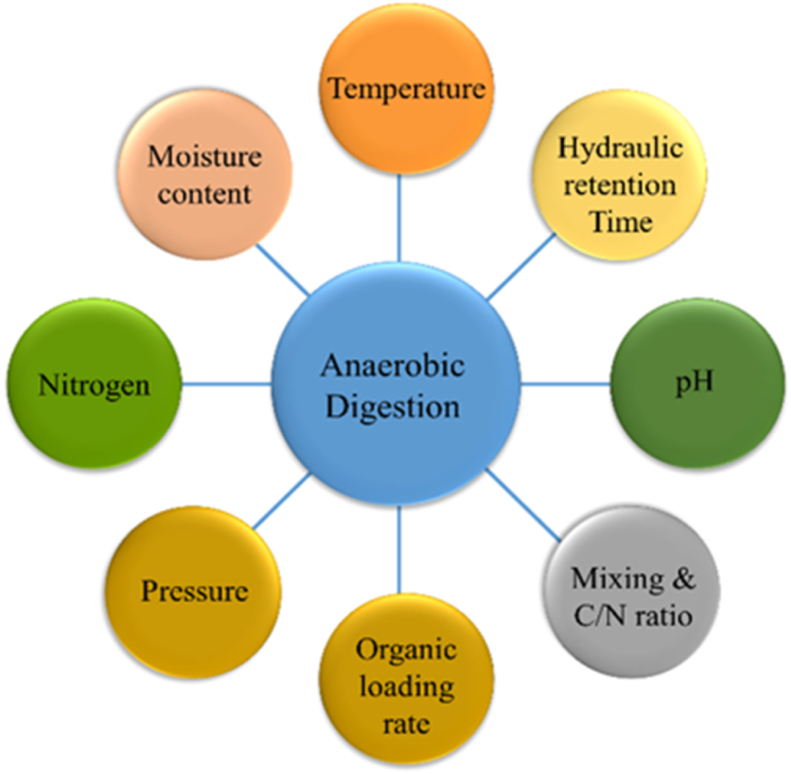
Schematic important factors influence the anaerobic digester.

**Fig. 6:**
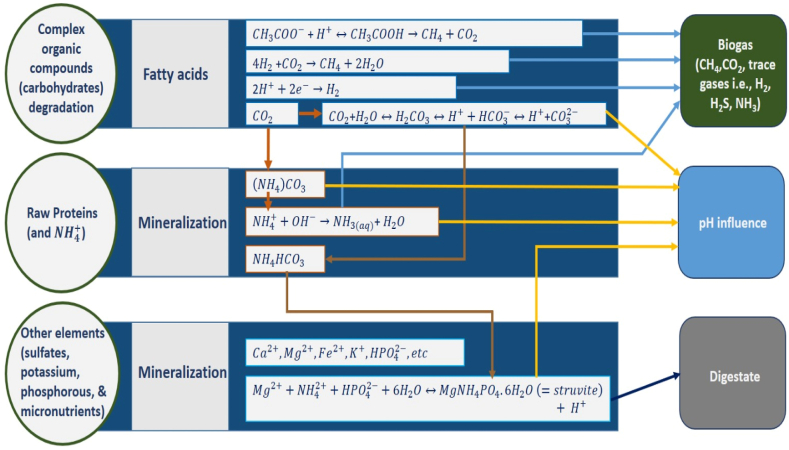
The influence of various ions originating from different degradation pathways on pH.

The degradation of organic material through AD is a multifaceted process that entails various degradation steps. The microorganisms involved in each step may have distinct environmental needs and, therefore, could be unique to that specific degradation stage. Fig. 5 depicts the important factors that influence the performance of AD (see Fig. 6).

### 7.1 Temperature

Temperature is a critical factor that impacts AD's overall performance; researchers have witnessed its pertinent effects on microbial populations, reaction kinetics, stability, and methane yield.^91^ Table 4 shows the various optimized temperatures that substantially altered the AD. The ideal temperature range for mesophilic AD typically varies between 35 and 40 °C, while thermophilic AD occurs at temperatures between 50 and 60 °C.^92^ The choice of temperature relies on various factors, comprising the kind of substrate, the desired digestion rate, and the desired quality and quantity of biogas produced. Studies have shown that temperature influences the configuration of the microbial community involved in AD, as different microorganisms have optimal growth rates at different temperatures. Furthermore, the temperature can affect the degradation rate of the organic matter and the production of methane and other desirable compounds. For example, thermophilic AD is typically faster and produces more methane per unit substrate volume than mesophilic AD. However, it also requires higher energy inputs for heating and may be more sensitive to fluctuations in operating conditions. Therefore, it is vital to consider the temperature conditions when designing and operating an AD system to ensure optimal microbial growth, energy production, and overall system stability and efficiency.^93^ Lowering the temperature during the AD process has been found to reduce microbial growth, decrease substrate utilization rates, and lead to a decline in biogas production.^94^ Furthermore, lowering the temperatures can result in energy depletion of cells, seepage of intracellular substances, and even complete cell damage. Conversely, high temperatures favor the generation of gases such as ammonia which significantly impedes the activities of the methanogenic bacteria and in turn, inhibits biogas production.^93^ Typically, AD is performed within the mesophilic temperature range.^95^ Operating within this range offers greater stability and requires less energy than other temperature ranges.^96^ The optimal operational temperature for AD is 35 °C with a digestion period of 18 days. However, even a smaller variation in temperature from 35 to 30 °C causes a decrease in the biogas production rate.^93^ A temperature range of 35–37 °C is optimal for efficient CH_4_ generation, and deviating from this temperature range causes a significant decrease in biogas production.^97^ The biogas production will resume once the necessary microbial populations proliferate. The thermophilic conditions have advantages such as rapid degradation rates of organic waste, increased production of biogas and biomass, decreased discharge viscosity, and significant pathogen removal rates.^98^

**Table 4:** List of factors affecting AD.

Parameter	Optimum value	Reference
Temperature (Mesophilic)	31–35 °C	^99^
Temperature (Thermophilic)	55–60 °C	(b), (e), 100
pH	6.8–7.20	(d), 101
C/N ratio	20–30	(a), 102
Retention time (Mesophilic)	20–30 days	(e), 103
Retention time (Thermophilic)	15 days	99e
Volatile fatty acid	<4000 mg L^−1^	^104^

### 7.2 pH

Researchers have shown that a pH of 7 maximizes the yield of biogas by favouring the growth of methanogenesis bacteria.^105^ Table 4 displays the various pH ranges that have significantly impacted AD. It was noted that a narrow pH range of 7–7.20 is suitable for bioreactors with added industrial sludge during the final 50 days of anaerobic incubation.^92^ Similarly, hydrolysis and acidogenesis happen at pH levels of 5.5 and 6.5, respectively.^106^ The maximum H_2_ can be achieved by maintaining the initial pH of a bio-system at 5–9.^107^ Small variations in pH of about 0.5 can significantly impact microbial metabolism and reaction kinetics, affecting gas production. While AD can occur within a broad pH range of 5.50–8.50, methanogens are particularly sensitive to changes in pH and function best within a pH close to 7 ^108^. The survival of methanogenesis bacteria is severely affected if the pH falls below 6.30 or exceeds 7.80, and the overall process will fail.^108^ However, unlike methanogenesis, hydrolysis and acidogenesis can function optimally within a pH range of 5.50–6.50.^109^ If the VFAs produced during acidogenesis are not broken down (metabolized), pH can drop significantly to less than 3, leading to process failure. High alkalinity during hydrolysis favors the formation of total ammonium nitrogen (TAN) can also cause disturbances in the process. pH extremes of around 3 or lower and 12 or more can adversely affect acidogenesis and inhibit hydrolysis rate.^110^ During biochemical interactions, the formation of different chemical species, such as NH_3_, CO_3_^2−^, and CH_3_COO-, can significantly affect pH variation in the digestate. For instance, the formation of ammonium carbonate ((NH_4_)_2_CO_3_) or the reduction of CO_2_ from the digestate liquid can directly cause an increase in pH.^111^ The formation of basic cations in the digestor, such as Mg^2+^, Ca^2+^, and K^+^ and the multivalent anions like SO_4_^2−^ and Fe(OH)_3_ reduction causes imbalances in the electric charge of the digestate liquid, which in turn causes an increase in pH. Similarly, a high organic loading causes the accumulation of carbonates such as CaCO_3_ or fatty acids also an important factor leading to lowering of pH. Kovács et al. (2015) demonstrated that protein-rich meat extracts tend to have reduced buffering capacity at higher organic loading inputs, leading to a drop in pH.^112^ The influence of variety of ions devising from different degradation pathways on pH is depicted in Fig. 5.

### 7.3 Moisture content

Moisture content (MC) is a crucial factor in the AD process, affecting the microorganisms' activity and the biogas production rate. High moisture levels generally facilitate the process, but it can be challenging to maintain consistent moisture levels throughout the entire cycle.^108^ As the digestion progresses, the initial high-water content gradually decreases to a lower level. However, the high water content can still impact the process by dissolving readily degradable organic matter. Researchers have reported an optimal range of 60–80% humidity can maximize methane production rates.^113^ A study examining AD for methanogenesis processes at different moisture levels (70% and 80%) revealed that the methanogenic phase initiated nearby the 70th day regardless of the moisture level.^42^ This indicates that moisture content can significantly affect AD performance and that the ideal moisture level may vary based on specific process conditions. Additional investigation is required to validate the effects of the moisture content on other elements of the AD process. It was proposed that a moisture level of about 70% may be ideal for methane synthesis. Notably, both moisture regimes resulted in comparable ratios of Biochemical oxygen demand (BOD) to chemical oxygen demand (COD), indicating similar organic matter removal efficiencies.^42^ This implies that moisture level is the key variable that dictates the efficiency of AD and in turn the biogas production.

### 7.4 Substrate/carbon source

The effectiveness of AD is heavily influenced by the type of substrate being employed. The process's efficiency is determined by microorganisms' ability to break down the organic matter in the substrate, with each microorganism having different preferences for the types of organic matter they can metabolize. More complex organic compounds like lignin and cellulose are harder to degrade and may require longer retention times. In comparison, simpler compounds like sugars and amino acids can be broken down more quickly, leading to faster biogas production rates.^114^ Furthermore, different types of carbon sources can support only specific groups of microbes, and the substrate's composition can impact the rate and efficiency of AD. Therefore, it's essential to characterize the substrate before digestion to determine its composition, including its carbohydrate, lipid, protein, and fibre contents. This information can be utilized to optimize the conditions for AD and maximize biogas production.^115^ Carbohydrates are the easily degradable organic components of municipal solid waste and have a high prospect for CH_4_ production under anaerobic conditions. Other organic components, such as lipids and proteins, are generally considered more recalcitrant and require longer digestion times and specific microbial populations for efficient degradation.^42^ Starch is a commonly available and low-cost substrate that readily undergoes hydrolysis to produce glucose, which in turn is fermented by microorganisms to yield methane. Starch can produce more methane than other common substrates like sucrose. It can be derived from various sources, such as corn, wheat, and potatoes, making it a versatile and widely available substrate. The substrate's initial concentration and total solid content are significant in AD. In general, a higher initial concentration and total solid content can lead to a higher methane production rate, but it can also increase the risk of process failure due to inhibition caused by high organic loading or high concentrations of NH_3_ and volatile fatty acids. Whereas, a lower initial concentration of substrate will take longer times to degrade the total solid content and this process yields lower methane yields. Here, lower substrate concentration influences the diversity and activity of the microbial population, significantly affecting the efficiency of the digestion process. It is therefore important to optimize the initial concentration and total solid content of the substrate for each specific case to maximize methane production while ensuring process stability.^42^

### 7.5 Nitrogen

Microorganisms in AD primarily require nitrogen as a nutrient for protein synthesis.^116^ During AD, the nitrogenous compounds present in organic waste, typically proteins, undergo a conversion process to form ammonium.^117^ Ammonium, resulting from the breakdown of nitrogenous compounds during AD, plays a vital role in stabilizing the pH value within the bioreactor. Microorganisms utilize ammonium as a nutrient source to produce new cellular material. For optimal mechanization, a nutrient ratio of C: N:P:S at 600:15:5:3 is generally considered adequate.^118^ At concentrations above approximately 100 mM, high ammonia levels can inhibit the biological process and lead to the inhibition of methanogenesis.^118^ The presence of ammonia in AD can influence hydrogen production and the removal of volatile solids, while small increases in ammonia nitrogen have little effect on total biogas production, higher increases can lower biogas production by 50% of the actual rate. The presence of excessive nutrients in AD beyond the acceptable limits has been found to restrict the AD process and decrease its overall performance. In essence, choosing a balanced carbon-to-nitrogen ratio, blending different feedstocks, introducing nitrogen supplements, and optimizing microbial activity ensures the desired optimal ratio of nutrients for enhanced biogas production.

### 7.6 Pressure

Research on the impact of pressure on AD has been limited. Though AD typically occurs at atmospheric pressure, owing to a series of reactions, there will be a build-up and exchange of gases in the reactor headspace, in turn, causes an over or under pressure on the liquid surface. Studies have suggested that lower pressures on the liquid surface resulted in enhanced biogas yields, and vice versa due to the solubility of CH_4_ in the liquid at higher pressure.^119^ The hydrostatic pressure level within the digester can also affect methane production. Past experiments have demonstrated that methanogenesis activities were highest at a digester depth of 4–5 m (400–500 mm H_2_O) for digesters over 10 m in height.^120^ One approach to improving methanogenesis is reducing hydrostatic pressure by transitioning from a vertically oriented digester to a horizontally oriented one. In laboratory-scale studies, pressurized anaerobic reactors have been developed and investigated, which can result in improved biogas yields by increasing CO_2_ solubility and stripping CO_2_ from the biogas, resulting in a higher concentration of CH_4_. However, an increase in pressures in the anaerobic reactor has not significantly increased biogas yield.^42^ The pressurized reactor concept has associated drawbacks, including technical challenges with leakage in the reactor system, pH reduction, and high investment costs.^42^

### 7.7 Organic loading rate (OLR)

The organic loading rate (OLR) is defined as the amount of organic material added per day per unit volume of the digester and is an essential parameter in AD. It is measured as the mass of volatile solids added per day divided by the volume of the reactor. Adjusting the OLR intentionally can significantly impact the digestion level for different influent inputs. In AD, a higher operating OLR is preferred because it promotes the enrichment of bacterial species, reduces the size of the reactor, lowers heating requirements, and reduces investment costs.^121^ To achieve a high OLR, various reactor configurations have been explored to reduce the hydraulic retention time (HRT). This can be done by diluting the substrate with or circulating the digestate into the main reactor.^122^ The achievement of a high organic loading rate (OLR) of 300 kg m^−3^d^−1^ was demonstrated in a lab-scale spiral automatic circulation (SPAC) bioreactor by reducing the HRT.^42^ The up-flow anaerobic fixed bed (UAFB) reactor type has also been found to allow for high operational OLR, but the maximum achievable OLR is lower than that of the SPAC type. It is important to note, however, that decreasing the HRT may result in microbial washout and eventual digester failure, as well as the possibility of volatile fatty acid (VFA) accumulation if the anaerobic digester is operated at a higher OLR or forced to run at a lower HRT.^123^ Methanogenic reactors are more stable when operated at a slightly higher HRT, usually between 10 and 25 days for continuous stirred-tank reactor (CSTR) configurations. Nonetheless, a recent study by Zhang et al. (2017) utilized a novel feeding strategy that maintained the OLR at a constant level while simultaneously reducing the HRT.^122^ Implementing this approach resulted in a reduction of ammonia nitrogen inhibition and an increase in methane yield. Microbial management is among the approaches currently being researched to optimize OLR and enhance biogas production, and the use of additives is gaining popularity as a potential strategy to achieve these goals.^124^ Table 5 exhibits a range of OLR applied in recent AD processes. In a study investigating the impact of OLR on the generation of biogas, methane yield, total volatile fatty acids (TVFA), alkalinity, ammonium, volatile solids (VS) removal efficiency, and process stability during the mesophilic semi-continuous AD of swine waste, the researchers reported that increasing the OLR (L^−1^ d^−1^) in the range of 2–5 g VS resulted in a proportional increase in the VS removal rate.^125^ In summary, regular monitoring and adjustment of feedstock mixture, feed rate, and supplementing the nutrients favours the microbial activity in turn the organic waste digestion. It is very important to gradually increase the organic loading rate to promote microbial population to grow, else a sudden loading will have negative impact on the biogas production (see Table 6).

**Table 5:** A range of organic loading rates (OLRs) applied in the recent AD process.

OLR	Feeds	Objective	Methane yield (wt.%)	References
0.4–3.1 kg COD/m^3^	Rice straw and pig manure	Co-digestion	Maximum (0.46 LCH_4_/gCOD_removed_) at OLR of 2.5 kg COD/m^3^	^126^
2.5 kg COD/(m^3^·day)	Rice straw (RS) and pig manure (PM)	Co-digestion	RS and PM's maximal methane production rates were 0.46 day 1, 350.79 mL (g-VS), and 45.36 mL (g-VS) day 1, respectively.	^127^
1 to 4 gVS/L/d for methane reactor; 3 to 12 gVS/L/d for H_2_ reactor	Laminaria digitata and micro-algae Arthrospira plantensis	Co-production of H_2_ and CH_4_	At an OLR of 2 gVS/L/d, methane production peaked and started declining. H2 production peaked at 6 gVS/L/d OLR.	^128^
30, 60, and 90 gVS/L	Sweet potato vine and animal manure	Co-digestion Performance	At OLR of 30 gVS/L and 60 gVS/L, methane yield for all co-digestion types was at its highest.	^129^
0.4 to 0.7 gCOD/L/d	waste Slaughter house	Pilot scale two-stage	With higher OLR, methane output fell.	^130^
4.6 and 8.6 kgCOD/m^3^/d	Food waste	Effect of feeding with or without dilution	At an OLR of 8.6 kgCOD/m^3^/d, the greatest methane productivity was 2.78 L/L/d; however, the system was unstable.	^131^
2.0 to 6.0 gVS/L/d	Food waste and horticulture waste	Comparison between two-stage and co-digestion AD	OLR rose for two-stage and co-digestion reactors, resulting in a drop in methane production.	^132^
Reactor ASBR: 0.93–25.0 gCOD/L.d Reactor AMBR: 1.04–19.65 gCOD/L.d	Composting leachate	Effect of OLR and series reactor AD	OLR >18.52 gCOD/L.d decreased biogas production with a maximum biogas yield of 10.08 gCOD/L.d.	^133^
1.12 to 3.88 kgCOD/m^3^/d	Beverage waste and sewage sludge	Co-digestion	The methane production kept rising until it reached an OLR of 2 kg COD/m^3^/d. OLR >3.8 kgCOD/m^3^/d inhibits the synthesis of methane.	^134^

**Table 6:** Selected reactor arrangements and their operational characteristics.

Reactor Types	Application	Optional parameters	Outputs	Reference
CSAB	Biohydrogen production	ca 1 L, Sucrose, inoculum heat shock, 40 °C, 6.6 ± 0.2, 30 to 40 gCOD/L/h, 0.5–6 h	Max. **HPR**: 15 L/L-h, Optimal HY: 3.5 mol/mol-sucrose	^164^
AnMBR	Biogas generation	2-phase (7 L and 20 L), Cheese whey, 37 ± 2 °C (both phases), 6.50 at the start (acidogenic), Max. 19.78 gCOD/L-d (methanogenic), 1 d and 4 d	**MC:** Max. 70% (methanogenic); biogas production exceeded 10 times reactor volume increased with OLR	^165^
BSAR	Biogas generation	1 L (5 units, equal volume), Pig manure (PM) and grass silage (GS), 35 °C, 6.5 to 8.0, 5 p.m.: GS (1:1, 1:3, 3:1, 1:0, 0:1), 90 days	Max. MY: 304.20 mL/gVS (at OLR 3:1 for PM: GS) Max. Cumulative MY: 8517 L (at OLR 3:1 for PM: GS)	^166^
CSTR	Methane production	5 L, CM and Laminaria Digitata, 35 ± 2 °C & 50 ± 1 °C, 8.0 ± 0.3, 2.5 to 2.9 gVS/L/d, 22 days	**MY**_avg_.: ca 225 L/kg VS (meso), ca 170 L/kg VS (Thermo)	^167^
ABSR	Biohydrogen production	3 L, Sucrose, 37 °C, 5.5 ± 0.2 (adjusted), 10–30 gCOD/L, 8 h	**HPR:** 10.9 ± 1.5 L/L-d, **HY:** 1.7 ± 0.2 mol/mol-sucrose	^168^
UASB	Hydrogen and methane production	kg/m^3^-d (biohydrogen reactor) and 2–10 kg/m^3^-d (methane reactor, Short (N/G)	L/kg COD fed (at OLR:25 kg/m^3^-d), Max. MPR: 0.91 L/L-d (at OLR: 8 kg/m^3^-d), Max. MY: 115.23 L/kgCOD (at OLR: 8 kg/m^3^-d)	^169^
SAnMBRs	Biogas generation and wastewater treatment	6 L (3 units), Synthetic low-strength wastewater, 25–30 °C, 7.0 ± 0.5 (adjusted), 1.1–1.65 kg COD/m^3^/day, 8–12 h	Max. **MPR**avg.: ca 2.9 L/d (HRT: 8, SRT: infinitive), Max. MYaverage: 0.29 L/gCOD (**HRT**: 8, SRT: infinitive), Max. specific **MY**: 0.068 L/MLVSS/d (HRT: 12, SRT: infinitive)	^170^
AGSBR	Biohydrogen production	ca 0.9 L, Glucose, 40 °C, 6.5, 20 gCOD/L, 4, 2, 1 and 0.5 h	HC: 36–41%, HY: 1.4–31.5 mol/mol-glucose	^171^
AFBR	Biohydrogen production and waste water treatment	ca 4 L, Synthetic wastewater, 37 °C, 4 (adjusted), 10 g L^−1^, 0.5–4 h	Max. HPR: 2.36 L/L-h, Max. HY: 1.16 mol/mol-glucose.	^172^
Semi-continuous	Process performance and digestate valorization	Mixing ratio: 1:0.7:0.3, C/N ratio: 13, OLR:2.7, SRT:30,	Co-digestion of dairy manure results in an approximately 2-fold increase in NH_4_^±^-N compared to mono-digestion.	^173^
CSTR	Biogas	Mixing ratio: 1:2.1, C/N ratio: 25, OLR:2, SRT: 21,	Increased methane production from swine dung when compared to mono-digestion	^68^

CSAB: anaerobic sludge blanket reactor, AnMBR: anaerobic membrane reactor, BSAR: batch system anaerobic reactor, CSTR: continuously stirred tank reactor, ABSR: anaerobic baffled stacking reactor, UASB: up-flow anaerobic sludge blanket reactor, SAnMBRs: submerged anaerobic membrane reactor, AGSBR: agitated granular sludge bed reactor, AFBR: anaerobic fluidized bed reactor.

### 7.8 Hydraulic retention time (HRT)

HRT increases treatment performance by influencing the relationship between substrates and bacteria. Shortening HRT is advantageous since it primarily addresses decreased capital expenditures and increased process efficiency.^135^ Table 4 shows the various optimized HRTs that substantially altered the AD. According to some reports, HRT must last at least 10 days to keep bacteria from being rinsed away. Prolonged HRTs were found to be more effective in producing biogas and methane when compared to lesser HRTs.^136^ Therefore, the design of AD systems is strongly influenced by hydraulic retention time (HRT) and solid retention time (SRT). The former pertains to the liquid phase's duration in the system, while the latter refers to the duration that the microbial culture is retained within the digester. HRT and SRT are equal when feedstock and mixed microbial cultures are present in the same phase. However, for substrates such as primary sludge and waste activated sludge, the interaction between solids and microbial cultures is biphasic, leading to HRT and SRT being distinct. SRT and HRT are similar for feedstocks such as food waste, kitchen trash, and municipal solid waste that is susceptible to AD.

The selection of HRT typically varies based on factors such as feed composition, processes, reactor volume, and temperature. Substrates high in starch and sugar content can be more degraded easily and thus require shorter HRTs. However, for substrates that are rich in lignocellulosic materials, such as agricultural residues, longer HRTs are typically required to ensure complete digestion. Additionally, larger reactor volumes may necessitate longer HRTs to enable sufficient contact time between the feedstock and microbial cultures. The specific processes employed in the AD system and the operating temperature can also influence the optimal HRT.^42^ Higher temperatures in the reactor can increase the decomposition rate, leading to faster digestion and shorter HRTs. Since thermophilic reactors operate at higher operating temperatures, they have lower HRTs than mesophilic reactors. Ultimately, the choice of HRT will depend on various factors, including feedstock composition, reactor size, temperature, and desired level of biogas production.^137^ Hence, the ideal operational Hydraulic Retention Time (HRT) typically falls within the range of 10–25 days, as it is neither too lengthy nor too short for most cases.

### 7.9 Ammonia

The feedstock undergoes protein and urea degradation to produce ammonia, which exists in two forms in the aqueous phase: ammonium ions (NH^4+^) and free ammonia (FA) or unionized ammonia (NH_3_). These two forms are collectively called total ammonia nitrogen (TAN).^117^ During AD, a portion of the organic nitrogen (Kjeldahl nitrogen) is biodegraded to inorganic ammonia. However, this degradation is only partial, and the proportion of organic nitrogen converted to inorganic ammonia ranges from 34 to 80%.^138^ Mostly NH_3_ is produced during hydrolysis, and its yield is influenced by various factors, such as temperature, pH, inoculum, or microbial community.^139^ Free ammonia, strongly inhibits methanogens via permeating cells, causes pH imbalance and in turn disruption of enzymatic reactions.^140^ When both temperature and pH levels are high, the ammonia solubility decreases, causing the dissociation equilibrium of aqueous NH_3_ to move towards free ammonia production, which dominates over ionic ammonia production. At mesophilic temperatures with a pH of 7, only about 1.25% of TAN is converted to free ammonia (FA). However, at the same temperature with a pH of 8, approximately 11.25% of TAN is transformed into FA, suggesting methanogens were subjected to ten times more toxicity at a pH of 8 compared to a pH of 7.^141^ The extent to which methanogens are affected by ammonia levels depends on various factors, such as the bacterial type and environmental conditions. Acetoclastic methanogens are more susceptible to high ammonia concentrations,^142^ while other research has shown that hydrogenotrophic methanogens are less tolerant.^143^ Studies have shown that some strains of acetoclastic and hydrogenotrophic methanogens can tolerate Total Ammonia Nitrogen (TAN) concentrations greater than 10 g L^−1 42^. When exposed to high ammonia levels, such as concentrations greater than 3 g L^−1^, a transition from acetoclastic methanogenesis to syntrophic acetate oxidation was observed, resulting in a 50% decrease in methane production.^42^ In terms of environmental factors, thermophilic methanogens are typically better equipped to withstand higher levels of free ammonia compared to their mesophilic counterparts; however, it was discovered that the process was unstable and susceptible to inhibition.^144^ Thus, to achieve optimal AD, it is essential to carefully select process conditions, including operating temperature, pH, type of inoculum, and feedstock. It is recommended in the literature to maintain the level of free ammonia below 0.2 g L^−1 117^. There are several physical and chemical techniques available to reduce free ammonia levels and mitigate ammonia toxicity, including air stripping, zeolite utilization,^145^ membrane filtration, and adjusting the C/N ratio.^146^

### 7.10 Volatile fatty acids (VFA)

VFAs, which are significant compounds produced during AD, are composed mainly of acetic acid/acetate, propionic acid/propionate, butyric acid/butyrate, valeric acid/valerate, caproic acid/caproate, and enanthic acid/enanthate.^147^ These intermediate compounds play a crucial role in altering AD and achieving superior stability in CH_4_ gas production and also synchronize the series of biochemical reactions in the AD process. However, VFA formation can be disrupted by various operational disturbances, such as changes in pH, temperature, organic loading rate, pH, and partial pressure of H_2_, which may result in non-optimal VFA formation. Accumulation of VFAs can also be toxic and even inhibitory to the AD process, depending on the types and levels of VFAs present. Table 4 displays the different optimized VFAs that can significantly impact AD. To maintain the high potential methane production and increase overall AD efficiency, it is not advisable to accumulate high concentrations of VFAs of any type in a well-controlled AD system. Various measures have been proposed to facilitate the speedup of the conversion of VFAs into CH_4_. These measures include selecting a suitable reactor type, optimal operating temperature, optimal pH, appropriate partial pressure of hydrogen, organic loading rate, and chemical add-ons.^148^ A pH of 8.0, dissolves 99.90% of the VFAs, whereas at pH 6.0, only 90% are dissolved.^149^

The choice of reactor design significantly impacts the conversion of VFAs. Multistage reactors are often more effective than single-stage reactors (SSR) because they allow for the ideal interaction of diverse groups of bacteria in each AD phase, leading to a balanced exchange of products and reactants, which results in increased VFA conversion. However, the high complexity of the design and the associated capital costs make two-stage reactors (TSR) less appealing for many real-world applications. Instead, researchers have focused on optimizing the design of single-stage reactors to maximize VFA reduction. Some promising examples include installing baffles in the reactor and using membrane bioreactors to accelerate the degradation of VFAs.^150^ Further research is required to lower the cost of multistage reactors or integrate more advanced features into single-stage reactors to optimize VFA utilization and improve AD efficiency. Temperature is another critical factor that affects VFA production and utilization. Microbial species' presence and growth rate are heavily influenced by temperature changes, which, in turn, affect their communication, resulting in the production and conversion of VFAs.^2^ Studies have investigated the impact of different temperature ranges (psychrophilic and mesophilic; 4–20 and 20–50 °C, respectively) on VFA production and composition during hydrolysis.^2^ Researchers have shown that a temperature rise enhances the rate of hydrolysis, resulting in increased solubility of carbohydrates and proteins and, in turn, a speed-up in VFA production. The effect of temperature is insignificant on the production of VFA; for instance, a decrease in acetate production from 55 to 43% when the temperature rises from 4 to 14 °C.^151^ Researchers also investigated the synergetic effect of temperature changes and feedstock pre-treatment on VFA conversion. The results of these studies varied but indicated that VFA conversion is influenced by temperature, feedstock, and pre-treatment techniques, all of which affect microbial populations and how VFAs are produced and consumed for biogas production. Moreover, it has been suggested that pH level influences the diversity and abundance of microbial communities in an anaerobic reactor, which can ultimately affect the production and utilization of VFAs. For instance, a study reported that the change in pH from 7.5 to 8.5 increased the relative abundance of acetate-utilizing methanogens, resulting in enhanced methane production and decreased VFA accumulation. Therefore, optimizing the pH level in anaerobic reactors can be critical in regulating the VFA concentration and improving the overall AD efficiency. Likewise, low partial pressure of hydrogen and balanced organic loading enhances the biogas production rate. Overall, various factors, such as reactor design, temperature, and pH level, can significantly affect the production and utilization of VFAs in AD. Future research in this field can focus on developing new strategies to optimize these factors for efficient VFA conversion, identifying new microbial communities for VFA degradation, and exploring novel pre-treatment techniques for improving VFA production. This study highlights the importance of monitoring the pH changes and taking necessary steps to maintain the optimal pH level, improving the conversion of VFAs into biogas. Therefore, it is essential to maintain the pH level within a suitable range of 6.5–8.5 for optimal biogas production from AD systems.^152^

### 7.11 Mixing

The degree of mixing can significantly influence an AD system's cost and proficiency. Here mixing promotes a uniform temperature across the digester, by promoting effective contact between microorganisms, substrates, and nutrients. Adequate mixing can also aid in reducing sedimentation and foaming occurrences, which can be caused by factors such as fat floating with adhering gas bubbles or filamentous microorganisms.^153^ While there are some conflicting results, reactors aided with mixing generally produce a higher yield of biogas than those without.^153^ Various mixing techniques, such as mechanical mixing with an impeller, hydraulic mixing through liquid recirculation, and pneumatic mixing by recirculating gases, can be applied at different frequencies (continuous or intermittent) and intensities (gentle or intermittent) and rigorous rotation speeds) to facilitate mixing. In full-scale AD with continuous mixing, up to 50% of the total plant energy can be consumed, with the mixer motor start-up requiring an additional 2.50% energy.^154^ Mixing strategies should be chosen based on the specific feedstock type and AD technology to optimize energy usage and increase plant productivity. Switching from continuous to intermittent mixing modes can reduce energy demand by over 25% while maintaining high plant productivity.^155^ Additionally, innovative technologies like gas-lifting reactors that use biogas's rising tendency for partial recirculation and digester agitation can provide the benefits of mixing without the need for a mixer. This technology has resulted in amended plant economies.^156^

### 7.12 C/N ratio

The critical role played by the C/N ratio of organic material in AD is highlighted, as demonstrated in Table 4, which indicates various optimized C/N ratios that can significantly impact the process. Unbalanced nutrients are a limiting factor for the AD of organic waste, which can be addressed through the co-digestion of organic mixtures to enhance nutrition and C/N ratios. However, it is advised to combine fish waste, wastewater from slaughterhouses, waste-activated sludge, and fruit and vegetable waste to properly balance the C/N ratio.^146^ This process has the advantage of buffering the organic loading rate and reducing anaerobic ammonia production from organic nitrogen, in turn, mitigates the limitations of fruit and vegetable waste digestion. A C/N ratio of 20–30 is generally considered sufficient to provide the necessary nitrogen for the process.^157^

### 7.13 Reactor types

The design of an AD reactor greatly influences the way feedstock is converted. Wet substrates (TS<15%) and high-solid substrates (15%<TS<40%) are generally processed using a wide variety of reactor designs. Table 2, summarizes the various parameters that influence the various types of reactors. The recent improvements include modifications in the process and reactor system, and feedstock tuning to meet the definite requirements of the product.^42^ It is expected that further developments will occur. There are two main types of digesters: batch and continuous. Batch digesters are simple, require fewer parts, and are less expensive. However, they are usually only used to determine the methane potential of substrates, as they require a long period of AD until the theoretical maximum is reached. Continuous digestion systems, on the other hand, produce gas consistently and are, therefore, more desirable in real-life scenarios. The AD systems can also be classified as single and multiple stage digestors. In a single-stage digestor, all the desirable reactions take place in a single reactor vessel. Despite the availability of multi-stage reactors, single-stage reactors remain the most widely used in AD applications due to their simplicity in design and lower costs.^158^ This configuration has been used to treat a variety of substrates, such as food waste, sludge, vegetable waste, municipal solid waste, bio-waste, and livestock manure, with process performance being optimized in many cases.^42^ For example, employing a single-stage reactor (such as CSTR, PFR, UASB, ASBR, and TPAD) has been shown to achieve optimal methane recovery when treating livestock manure, and this can be further enhanced by circulating back the process liquid into the reactor.^42^^,^^159^ Recirculating digestate leads to increased liquid retention time and decreased washout of microbes during digestion. In a two-stage system, the feed is charged into the acidogenic reactor, where the material undergoes hydrolysis, acidogenesis, and acetogenesis. Partly digested materials are then transported to the methanogenic reactor, where CH_4_ is formed. The two-stage process ensures faster and more competent biogas production than a single stage, with methane recovery from volatile solids exceeding 90% in some cases.^42^ However, due to their more complex design, two-stage reactors are generally more expensive to set up. The concept of a three-stage reactor design was developed in the early 1990s and involved several phases. In the first phase, feedstock undergoes semi-anaerobic hydrolysis at a low HRT, followed by removing undigested materials and transferring them to the second reactor, where acidogenesis occurs. Finally, in a three-stage system, the output liquids and solids are directed to a tertiary reactor, which produces methane-rich biogas. Similar to the two-stage system, the discharge from the tertiary reactor can be recirculated back to the digester, depending on the desired hydraulic retention time and process efficiency.^160^ A three-stage reactor achieved over 95% COD removal efficiency for food waste AD ^160^ Recently, another study reported an 11–23% increase in methane yield for a three-stage co-digestion of food waste and horse manure.^161^ In addition to the above configurations, AD systems have undergone various amendments in recent decades to enhance the retention efficiency of slow-growing methanogens. The development of the Up-flow Anaerobic Sludge Blanket (UASB) reactor, where a dense sludge bed allows for the retention of methanogenic sludge within the reactor, has led to significant improvements in retention efficiency.^162^ Various UASB reactors are used to treat waste and wastewater effluents with varying characteristics. The internal circulation (IC) reactor is highly efficient due to its feasibility, robust resistance to external accidents, ability to handle high organic loading rates, and low investment cost.^42^ Another successful technique is using membrane bioreactors (MBR), which can effectively retain active biomass within the system by using a membrane to separate cells and inhibitory components. Though the MBR has numerous advantages, it has the inherent disadvantages such as high capital cost, membrane fouling, complexity in the maintenance, and membrane replacement costs. Gaida et al. (2017) have provided excellent documentation of the present advancements in sensor technologies for AD control.^163^

## 8 Techno-economic feasibility of AD

Economic viability, technological feasibility, and sustainability criteria can be used to evaluate a techno-economic analysis and life cycle assessment, which are essential for sustainable processes. Most companies and financiers are interested in using AD to generate power from biogas. Processes include the distribution of raw materials, the sorting of trash, the types of feedstock, and profit concerns frequently view it as a high-risk investment.^174^ Local governments promote research by providing funds and permitting businesses for this sector. Techno-economic analysis and life cycle assessment provide insight into the AD system's feasibility, which is crucial.^175^ Dereli et al. (2010) techno-economic analysis shows that the initial sludge digestion might yield 3.33 times as much power as the AD of sewage sludge and organic fraction of municipal solid waste (OFMSW) mixed.^176^ The amount of energy that the AD procedure for sewage sludge and OFMSW can produce is roughly 56,000 kWh/d, equivalent to the methane savings of nearly €1.5 million from the internal combustion engine and power production system. Bolzonella et al. (2006) claim that adding OFMSW to sewage sludge enhanced biogas generation by 240%.^177^ The average treatment cost per tonne of OFMSW was €50 at an average concentration of 50 tonnes per week. According to a life cycle study, the AD of OFMSW and sewage sludge is a sustainable waste management option for small-scale systems.^178^ According to Edward et al. (2017) life cycle analysis, AD has less potential to harm the environment than the present waste management system, including less potential for acidification, global warming, and fossil fuel depletion.^179^ The dearth of or a decrease in trash disposal fees can result in low returns. According to reports, the AD process can generate a sizable amount of cash and is financially viable. For instance, according to Moraes et al. (2014), AD of vinasse from sugarcane bio-refineries may benefit from an energy, environmental, and economic standpoint, optimizing the plants' sustainability.^180^ AD of algal biomass might generate around $10,6 million annually.^181^

## 9 Challenges, conclusions, and future perspectives of the AD process

Despite several advantages, bioenergy production from organic waste through AD is fraught with numerous significant challenges. Social acceptance of this process is also affected by environmental and health-related concerns. Biogas, which is produced during AD, contains a range of undesirable and potentially hazardous substances, including hydrogen sulphide (H_2_S), silicon (Si), volatile organic compounds (VOCs), carbon monoxide (CO), and ammonia (NH_3_).104b H_2_S and NH_3_ are highly corrosive and harmful gases that can cause damage to metal components and combined heat and power units.^182^ The presence of H_2_S can adversely affect the quantity and quality of the biogas produced and emit harmful emissions. In addition, it can corrode the biogas purification system.^183^ Due to impurities in biogas generated through AD, it typically requires pre-treatment to improve CH_4_ yields and post-treatment to remove H_2_S. However, these processes are energy-intensive and costly.^183^ Moreover, climate effects also influence biogas production, similar to other renewable energy sources. Some of the common strategies to mitigate the effect of harmful gases include gas scrubbing, digestate treatment, and desulfurization system. Combination of these strategy can effectively mitigate health and environmental impacts of hazardous gases emitted during AD.

The review claims that AD is a viable biological method for treating various solid organic wastes and sludge and converting them into valuable products. Co-digestion enhances the overall digestion of co-substrates by balancing the nutrients, which in turn increases biogas production and process economics. It is crucial to optimize variables including temperature, pressure, pH, moisture, C/N ratio, OLR, HRT, reactor types, substrate and co-substrate types and quality, and ambient conditions to maximize the efficiency of the AD process. Advanced molecular techniques such as microbial structure, function, and ecological relationships also play a significant role in improving the digester's performance. These techniques enhance our understanding of the AD process by revealing complex details such as microbial structure, their function, and their interaction with different microbial communities. This information enables researchers to make viable decisions to optimize the performance of AD for several applications such as treatment of OSW and production of biogas. This technology holds enormous potential for future environmental and agricultural sustainability applications, with energy production as an added advantage. It is crucial to conduct techno-economic studies of AD to utilize this technology appropriately and gain its inherent benefits.

The biogas from AD has several benefits over other renewable energy solutions. It can be produced on demand, stored easily, transported with existing gas pipelines, and utilized in the same ways as that of natural gas. Additionally, biogas uses sustainable sources of energy and heat while replacing fossil fuels in transportation. However, the effectiveness of the AD process relies on the optimization of several factors. Policymakers must encourage and bring awareness about the advantages of solid waste management using AD; its products are to be used as green fuels instead of fossil fuels. With persistent efforts, biogas can be a novel solution to a spectrum of environmental problems. Utilizing biogas as a renewable energy source can moderate the dependency on fossil fuels and aid in the transition to a more sustainable energy system. In essence, Biogas has the potential to play a significant role in combating climate change and advancing sustainable development with the right process optimization and encouraging policies from governments and stakeholders.

## Ethical approval

The author does not have any ethical issues.

## Funding

The authors have no relevant financial or non-financial interests to disclose.

## Availability of data and materials

The datasets generated and analyzed during the current study are available from the corresponding author upon reasonable request.

## CRediT authorship contribution statement

Ranjeet Kumar Mishra: data curation, investigation, visualization, conceptualization, data curation, methodology, investigation, visualization, writing-original draft, and supervision. ; D. Jaya Prasanna Kumar, Sampath Chinnam and Prakash Binnal: Curation, conceptualization, visualization editing the original draft. ; Naveen Dwivedi: investigation, visualization, editing draft, and supervision.

## Declaration of competing interest

Author do not have any Conflict of Interest.
